# Circadian Clocks, Redox Homeostasis, and Exercise: Time to Connect the Dots?

**DOI:** 10.3390/antiox11020256

**Published:** 2022-01-28

**Authors:** Conor McClean, Gareth W. Davison

**Affiliations:** Sport and Exercise Sciences Research Institute, Ulster University, Newtownabbey BT37 0QB, Northern Ireland, UK; gw.davison@ulster.ac.uk

**Keywords:** circadian rhythms, reactive oxygen and nitrogen species (RONS), exercise training, antioxidant

## Abstract

Compelling research has documented how the circadian system is essential for the maintenance of several key biological processes including homeostasis, cardiovascular control, and glucose metabolism. Circadian clock disruptions, or losses of rhythmicity, have been implicated in the development of several diseases, premature ageing, and are regarded as health risks. Redox reactions involving reactive oxygen and nitrogen species (RONS) regulate several physiological functions such as cell signalling and the immune response. However, oxidative stress is associated with the pathological effects of RONS, resulting in a loss of cell signalling and damaging modifications to important molecules such as DNA. Direct connections have been established between circadian rhythms and oxidative stress on the basis that disruptions to circadian rhythms can affect redox biology, and vice versa, in a bi-directional relationship. For instance, the expression and activity of several key antioxidant enzymes (SOD, GPx, and CAT) appear to follow circadian patterns. Consequently, the ability to unravel these interactions has opened an exciting area of redox biology. Exercise exerts numerous benefits to health and, as a potent environmental cue, has the capacity to adjust disrupted circadian systems. In fact, the response to a given exercise stimulus may also exhibit circadian variation. At the same time, the relationship between exercise, RONS, and oxidative stress has also been scrutinised, whereby it is clear that exercise-induced RONS can elicit both helpful and potentially harmful health effects that are dependent on the type, intensity, and duration of exercise. To date, it appears that the emerging interface between circadian rhythmicity and oxidative stress/redox metabolism has not been explored in relation to exercise. This review aims to summarise the evidence supporting the conceptual link between the circadian clock, oxidative stress/redox homeostasis, and exercise stimuli. We believe carefully designed investigations of this nexus are required, which could be harnessed to tackle theories concerned with, for example, the existence of an optimal time to exercise to accrue physiological benefits.

## 1. Introduction

A diverse array of physiological functions, human behaviours, and social connections are determined by the complex interactions between the environment (light/dark cycles, temperature variations, and seasonal food opportunities) and endogenous biological, or circadian drivers. Such an intricate controlling mechanism is susceptible to disruption and, in current modern society, misalignment between the circadian system and environmental cues is a frequent occurrence associated with negative health consequences [1]. Modern chronic diseases are linked to changes in human lifestyle compared to our hunter-gatherer ancestors [2] involving, but not exclusively limited to, low levels of physical activity (PA) and exercise; prolonged sitting; regular access to highly palatable and energy-dense foods; inadequate/disrupted sleep quality/duration; shift work; and social jetlag [3]. These changes in contemporary work and domestic habits have outpaced genome adaption and the underlying circadian rhythms are thus exposed to dysregulation (or shifts) that can predispose to chronic disease [4].

Oxidative stress has long been implicated in the pathogenesis of several chronic lifestyle-related diseases (e.g., type 2 diabetes mellitus) [5] and the interplay between circadian clocks and oxidative stress is evident whereby: (i) disruption to circadian rhythms can alter redox homeostasis leading to oxidative stress and (ii) elevated production of reactive oxygen and nitrogen species (RONS) may induce circadian oscillations [6]. While still in its relative infancy, research on the effects of exercise on circadian rhythmicity seems to be encouraging; certainly not discouraging [7,8,9]. At the same time, certain exercise stimuli may evoke oxidative stress, but to the contrary, many exercise-mediated adaptations seem to occur via contractile-induced RONS signalling, acting in a manner that can be explained by the concept of hormesis and other multi-dimensional models [10]. Hormesis, in this context, is when a potentially harmful agent (e.g., exercise) provokes an adaptation to a damaging agent (such as RONS-disrupted signalling) to up-regulate, for example, enzymatic antioxidant capacity [11]. Hence, exercise appears to be a potent activator of oxidative stress, the circadian clock, and, at the correct dose (intensity and volume), elicits desirable health outcomes. As a clear bi-directional link has already been established between the circadian clock and redox homeostasis/metabolism, and that exercise appears to be a potent stimulus of both, we propose that further exploration of exercise, circadian rhythms, and redox biology combined may reveal further important insights in this intriguing field of investigation. In this perspective, the exercise paradigm may help unravel some emerging concepts such as the potential existence of an optimal time to exercise for health benefits. The positive effects of exercise on health may be partially mediated via changes in tissue molecular clocks and/or the outcomes may be modified depending on the timing (and intensity) when exercise is performed [12], but the role, whether directly or indirectly, that RONS and oxidative stress/redox signalling play in such responses remains largely unexplored. Therefore, given the emerging nexus of circadian and redox biology for health and disease prevention, this narrative review will examine the multifactorial interrelationship between circadian clocks and oxidative stress relevant to the important biological stressor, exercise.

## 2. Circadian Rhythms and Molecular Clock Control

**Figure 1:**
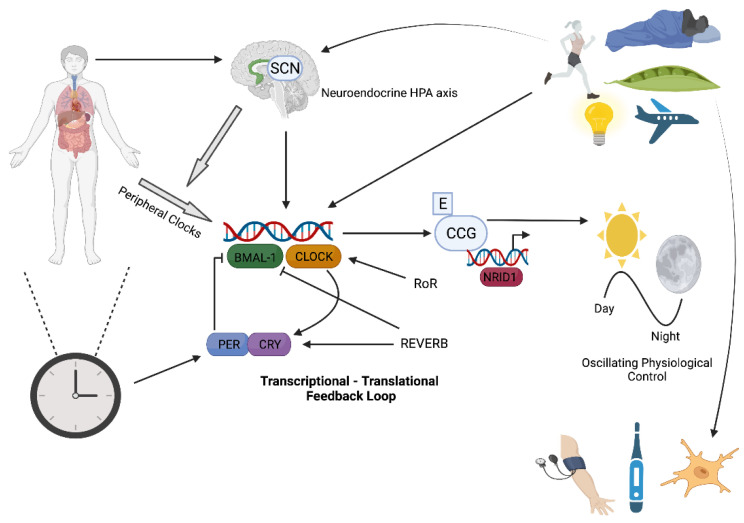
Circadian Rhythms in Humans. Crucial biological and physiological processes such as blood pressure control, antioxidant expression, body temperature, and immune function normally fluctuate in a circadian pattern (~24 h). These oscillations are coordinated by circadian clocks distributed in virtually every cell. The core molecular clock functions to direct a daily program of gene transcription and protein expression. The molecular clock involves a transcriptional–translational feedback loop (TTFL) of core clock genes (positive limb: *BMAL-1* and *CLOCK*; negative limb: *PERs* and *CRYs*) that act to modulate the gene expression of clock-controlled genes (CCG) which generate tissue-specific circadian rhythms in transcription and cellular function across the day, even in the absence of external cues. The principal pacemaker of the circadian clock in humans is located within the suprachiasmatic nucleus (SCN) region of the hypothalamus. This master clock coordinates the activity of downstream peripheral tissue clocks in response to several stimuli such as the day-night cycle, food ingestion, exercise, and sleep. Figure key = SCN: suprachiasmatic nucleus; HPA: hypothalamic-pituitary-adrenal axis; CCG: clock-controlled genes; E: E-box motif.

Apparent in virtually all forms of life, circadian rhythms are endogenous 24-h oscillations in behaviour and biological processes enabling organisms to physiologically adjust to the transitions between light and dark (day and night). The circadian clock drives fluctuations in a diverse set of biological processes including sleep, locomotor activity, blood pressure, body temperature, and blood hormone levels [13]. Driven by cellular clocks distributed across the body, these rhythms control mammalian adjustment by preparing the brain and other tissues to perform biologically appropriate functions relative to the anticipated day or night pattern [14,15]. The principal pacemaker of the circadian clock in humans, the so-called central clock, is located within the suprachiasmatic nucleus (SCN) region of the hypothalamus, functioning as an autonomous timekeeper that coordinates the activity of downstream peripheral tissue clocks [15]. Neurons of the SCN receive input from the retina, via the retino-hypothalamic tract, to ensure its molecular clocks are synchronised to light-dark cycles, known as photic entrainment [16]. The SCN can be stimulated by cues other than light, especially by serotonin and melatonin, although these are thought to be internal feedback regulators as opposed to primary circadian rhythm initiators [6]. Using neuro-endocrine actions involving melatonin, insulin, and glucocorticoids (via the HPA axis), as well as the autonomic nervous system [6,17], the SCN relays its coordinating prompts to peripheral clocks [12]. In turn, these peripheral clocks oversee localised, temporal gene and protein expression patterns required for several physiological processes and functions across the ~24-h period [15,17]. Beyond the autonomous cues from the SCN, mammals can also synchronise their inherent timing systems in response to fluctuations in other environmental cues (termed *zeitgebers*) such as changes in temperature and food availability [18], but light remains the strongest zeitgeber [6]—illustrated in Figure 1. While this innate flexibility to varying environmental stimuli is unquestionably important for adaptation and survival, it may also expose the host to negative consequences, such as the onset of sleeping and metabolic disorders, when the rhythms become misaligned or dysregulated [19].

The core molecular clock is a self-sustaining, transcriptional–translational feedback loop (TTFL) that exists in virtually every human cell and functions to direct a daily program of gene transcription and protein formation, permitting rhythmic adjustments in response to specific entrainment signals [12,13]. This molecular clock is comprised of a positive transcriptional limb and a negative feedback limb [16]; interrogation of this regulatory mechanism was recognized in 2017 when Jeffery Hall, Michael Rosbash, and Michael Young were awarded the Nobel Prize in Physiology or Medicine [13]. Their work, initially in the *Drosophila* model, identified some of the most important clock genes involved in the negative limb of the TTFL such as *Period* (*PER 1-3*) and *Timeless* (*Tim*; or *Cryptochrome (CRY*) as it is known in mammals). These were later followed by the identification of the positive limb transcription factors, CLOCK and BMAL-1 [20]. The heterodimerization of BMAL-1 and CLOCK, or its homolog NPAS2, initiates the circadian cycle. Once dimerized, BMAL-1 and CLOCK bind to E-box motifs in the promoters of target genes, initiating the intracellular transcriptional processes of various rhythmic proteins [6,16]. Two groups of these transcriptional targets are the PERs and the CRYs. The PER and CRY proteins, through the formation of a second heterodimer, translocate back into the nucleus, and interfere with the activity of BMAL1 and CLOCK at promoter sites, thus completing the cycle by inhibiting CLOCK/BMAL-1 transcriptional activity [6]. This core clock is refined to a 24-h period by the combined actions of several post-translational mechanisms (e.g., covalent histone modifications) carried out by a network of secondary clock proteins involving the kinase family [16,21]. An influential secondary loop driven by BMAL-1 activity involving the nuclear hormone receptors REVERB and ROR has also been identified. These proteins can inhibit and activate the transcription of the BMAL-1 gene (ARNTL), respectively, to ensure its rhythmic expression, and thus they can exert influence on circadian regulation [22]. In this loop, the expression of REVERB proteins serves to repress transcription in the promoter and enhancer regions of target genes, including ARNTL, whereas ROR competes to enhance ARNTL expression. A further CLOCK/BMAL1-driven sub-loop contains the PAR-bZip factors DBP, TEF, and HLF [23,24,25,26,27,28,29,30,31,32,33,34]. These compete with the repressor NFIL3 (or E4BP4), driven by the REV-ERB/ROR loop, to drive the expression of clock genes from D-box-containing promoters [22].

In addition to its timekeeping function via the TTFLs discussed above, the core molecular clock directly modulates the expression of over 4000 genes in 24-h cycles of transcription with diverse expression phases, resting with the combination of cis-elements (E-box, RORE, D-box) as the promoters and enhancers of specific clock-controlled genes (CCGs) [12,23,24]. These CCGs are expressed in virtually every cell and can generate tissue-specific circadian rhythms in transcription and cellular function, even in the absence of external cues. The circadian clock regulates between 10 and 50% of all transcripts in a cell, depending on tissue type, and influences critical processes such as cell cycle, redox homeostasis, inflammation, and metabolism [13]. The fact that nearly half of the mammalian protein-coding genome expresses tissue-specific circadian rhythmicity [13] underlines the importance of maintaining these oscillations and the seemingly critical role played by circadian rhythms in optimising cell and tissue function for normal health. In fact, cardiovascular function, energy metabolism, fluid balance, inflammation/immune function, cognition/neurological responses, and some of their biological drivers (CCGs and endocrine hormones) all appear to exhibit, either directly or indirectly, some degree of rhythmicity [25,26]. Beyond the central or core factors that directly control the core molecular clock, external factors can regulate the stability, phase, or function of core molecular clock proteins. Hypoxia (and fluctuating oxygen levels) acts on circadian rhythms (body temperature, metabolic rate, cortisol, and melatonin release in humans) through a number of mechanisms involving HIF-1α, with mounting evidence showing cross-talk between the HIF pathway and the circadian clock [27].

Disruption of the circadian system has direct consequences for human health by the uncoupling of multiple physiological processes and the disturbance of normal homeostasis, thus increasing the risk of several disorders and diseases [28]. In addition to genetic disruption to the clock circuit (via specific mutations), humans encounter several potent zeitgebers, often in simultaneous exposures, which hinder circadian regulation such as shift work, excessive artificial/night-time light exposure, disrupted eating patterns (often as a result of shift work), and international travel across several time zones (inducing jet lag).

The current understanding of the effects of circadian disruption has relied mainly on observational studies exploring occupational practices. Jet lagged cabin crew demonstrated temporary cognitive defects and structural brain changes following trans-meridian flights (across at least seven times zones) with five-day recovery intervals when compared to colleagues with fourteen-day recovery intervals [29]. Shift work and social jetlag also disrupt the circadian system and are associated with an increased risk of multiple diseases such as neurological disorders, diabetes, cancer, and cardiovascular disorders [30,31,32]. In addition to occupational requirements, recreational activities may also affect normal circadian rhythms. For example, the common use of light-emitting (via so-called ‘blue light’) electronic devices for reading, communication, and entertainment inhibits melatonin production which poses an increased risk for several circadian-related disorders when such devices are used in the evening [33]. As a consequence of modern living per se, many people are no longer exposed to the natural light/dark cycle of our ancestors. Humans are now inclined to be more active later into the night-time hours, often accompanied by eating and alcohol consumption, leading to a delayed bedtime but rising earlier than our natural rhythm would instigate. This is arguably leading to a chronic state of circadian disruption and associated risks [34].

## 3. Oxidative Stress and Redox Homeostasis

Humans are continually exposed to RONS from both endogenous (e.g., from aerobic metabolism and the immune response) and exogenous sources (e.g., tobacco smoke, UV light, etc.) and thus, antioxidants function to help prevent/curb high levels of oxidative stress and maintain redox homeostasis. Accordingly, aerobic organisms possess an intricate antioxidant defence system [35] that comprises an orchestrated synergism between several endogenous and exogenous antioxidants attempting to control the RONS produced in cells and tissues [36,37] which arise as a consequence of everyday activities such as the food we eat [38]. Oxidative stress is defined as ‘an imbalance between oxidants and antioxidants in favor (sic) of the oxidants, leading to a disruption of redox signaling (sic) and control’ [39]. Thus, the biological (physiological or pathological) effects of RONS depend critically on the amounts and location of production, relative to local antioxidant defences. For instance, when formed in low/moderate amounts, they can act as crucial mediators of signal transduction pathways e.g., in the growth of vascular smooth muscle cells (VSMC) and fibroblasts [40] and for exosome control and myokine release [41]. Yet, excessive RONS may cause widespread cellular toxicity [6,42]. Indeed, work from several labs, including our own, has demonstrated how RONS instigate oxidative damage by altering susceptible lipids, proteins, and DNA in human volunteers [43,44,45]. This can lead to impaired transcription and translational processes, altered protein function, and production of secondary by-products and metabolites which can further RONS production and/or cellular damage [46]. Although still an area of some discourse, RONS and other redox-active species have increasingly been acknowledged as fundamental regulators of genes, proteins, and the associated molecular signalling pathways integral to the regulation of many biological processes and functions [46,47].

### 3.1. Oxidative Stress, Redox Homeostasis, and Exercise

**Figure 2:**
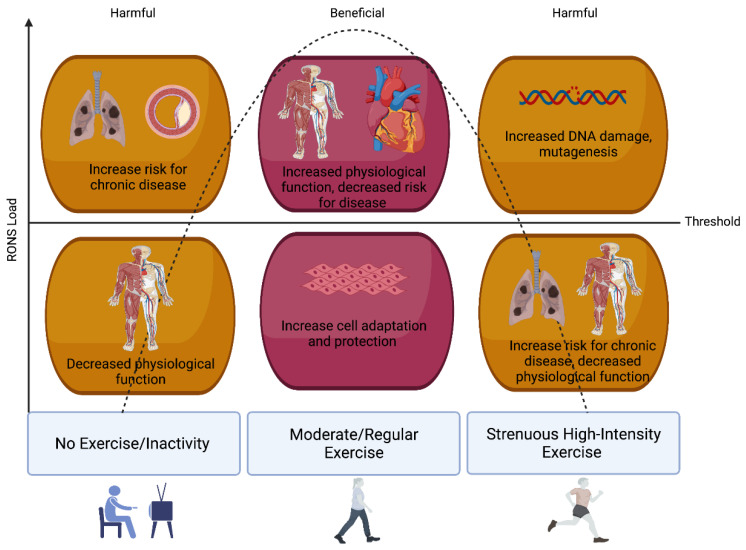
Exercise-induced RONS, Oxidative Stress, and Hormesis. The fundamental basis of the hormesis theory can be applied to exercise-induced RONS formation: low or transient increases in RONS, from moderate-intensity and regular exercise, activate signalling pathways that induce adaptive and protective responses. Whereas inactivity and/or sporadic strenuous/high-intensity exercise can lead to a RONS load that overwhelms antioxidant defences leading to oxidative stress, impaired physiological function, and an increased risk for chronic disease.

Multiple studies have now established a link between exercise (especially strenuous and/or exhaustive exercise) and oxidative stress [10,48]. The production of RONS in response to exercise, especially acutely, has long interested researchers and offers an intriguing model to examine the dynamic role of RONS from both the physiological and pathological perspectives—as depicted in Figure 2. The latter is important to note as evidence suggests that exhaustive (long duration) and/or strenuous exercise (high-intensity maximal exercise, marathons, triathlons, and overtraining) can induce detrimental, oxidative DNA alterations if left unrepaired [49,50]. Yet, during low or moderate-intensity exercise, the generated RONS may serve to act as signalling molecules responsible for the initiation of exercise and skeletal muscle adaptation [50,51,52], including accentuation in antioxidant enzymes and drivers of mitochondrial biogenesis [53]. In fact, the observation that low doses of stressors can exert beneficial effects, but when in excess can be toxic, forms the fundamental basis of the hitherto mentioned hormesis theory. This paradoxical effect has led to the extension of the hormesis theory to exercise induced RONS formation: transient increases in RONS can alter signalling pathways and/or cause molecular damage that induces adaptive responses that protect against subsequent stronger stress [54] such as that evident in human volunteers for the repeated bout effect following eccentric muscle contractions [55]. Moreover, when a single bout of exhaustive exercise is performed by well-trained human volunteers, a large elevation in oxidative damage is not observed [56]. Mounting evidence suggests that exercise-induced RONS are essential upstream signals for the activation of redox-sensitive transcription factors (e.g., Nrf2, AP1) and the induction of gene expression associated with exercise. As such, redox processes are increasingly recognized as an integral part of normal human biology and especially exercise (for further reading see the review by Margaritelis et al. [57]).

The adaptive potential of exercise and the extent of RONS production seems to be related to the exercise intensity of the bout or training stimulus [58]. High-intensity exercise produces higher concentrations of RONS than exercise at low/moderate-intensity aerobic exercise at <50% ${\dot{V}O}_{2\max}$ [59]. While we have recently reported that acute intense/prolonged exercise can induce oxidative DNA damage in human volunteers, this damage does not persist indefinitely and is most likely repaired, at least in trained/experienced athletes [10]. This damage might act as a trigger for repair to prime the cell for further, subsequent damage and thus underpin the adaptive process and promote longevity. For instance, master endurance athletes are shown to have a longer telomere length (TL), a marker of biological age, than non-athlete, age-matched controls [60,61]. In addition, three weeks of high-intensity interval training (HIIT) in humans improves plasma antioxidant capacity [62]. Furthermore, recent evidence in animal models has reported reductions in maximal exercise capacity, several exercise-responsive proteins, and mitochondrial network adaptations in mice lacking a functional NOX2 complex following a HIIT programme [62]. Thus, the fine balance between redox signalling and oxidative stress in the response to exercise requires further elucidation. A growing appreciation for the role of redox homeostasis has allowed researchers to understand that RONS can offer distinct signalling roles depending on where they originate. The following is a summary of the main sources of RONS production in contracting muscle and the corresponding antioxidants that converge to support redox balance.

### 3.2. Skeletal Muscle as a Biological Source of RONS

Several RONS are generated continuously in, or very close to, skeletal muscle both at rest and during exercising contractions [63]. Of these, O_2_^•−^ is the primary species. Early work revealed mitochondria as a source of free radical production at rest [64], but it appears mitochondria are not a major source of RONS generation in exercising muscle [65]. A number of other potential sources for primary and subsequent RONS (e.g., H_2_O_2_) have been identified and include NADPH oxidases (NOX; which is active across several cellular organelles), xanthine oxidases (XO), and phospholipase A_2_ (PLA_2_) [49]. It should also be noted that nitric oxide (NO^•^) production occurs both at rest and during/following exercise in muscle. Interestingly, via the convergence of NOX4 and two forms of neuronal NOS in mouse models, the resultant NO^•^ yields peroxynitrite (ONOO^−^) proposed to be involved in the signal transduction pathways for mTORC1 integral to muscle hypertrophy associated with strength/resistance training [66,67,68,69]. NO^•^ itself has also been postulated to confer an intrinsic effect on the regulation of insulin secretion, and glucose transport and metabolism, which are also accentuated via exercise [70,71].

The NOX enzymes are viewed as an important source of RONS in skeletal muscle, particularly the NOX2 and NOX4 isoforms [72]. NOX2 is located within the sarcolemma and T-tubule, whereas NOX4 is located in both the sarcoplasmic reticulum and the mitochondria [73]. NOX4 is constitutively expressed and postulated to be involved in basal RONS production, whereas NOX2, sensitive to several stimuli, is believed to be the primary source of NOX-mediated RONS production in contracting muscle [11,72]. Whether or not NOX enzymes are the major source of muscular RONS is debatable as complexities remain in measuring their activity [49]; although recent advances using genetically encoded redox probes offer attractive possibilities in this respect [11,63], further scrutiny is beyond the scope of this narrative.

### 3.3. Antioxidants

As RONS and redox reactions appear to be pivotal for cellular health and function, mammalian cells require an inherent regulatory control mechanism to sustain redox homeostasis and prevent damage from oxidative stress. The antioxidant network of enzymatic and non-enzymatic antioxidants maintains RONS at physiological levels, thereby maintaining redox control. The principal antioxidant enzymes include superoxide dismutase (SOD), catalase (CAT), and glutathione peroxidase (GPx) [74]. Important non-enzymatic or dietary antioxidants include ascorbic acid (vitamin C), α-tocopherol (vitamin E), carotenoids, and flavonoids. In addition to the three main groups of enzymatic antioxidants (SODs, GPx, and Catalase), other accessory antioxidant enzymes help maintain redox balance. The Thioredoxin (Trx) antioxidant system comprising of Trx and the enzyme Trx reductase, serves as an electron donor to drive antioxidant systems and regulate proteins (in dithiol-disulfide exchange reactions) in response to a changing redox environment. This antioxidant assists in general metabolism (including DNA synthesis) and the prevention of deleterious levels of oxidative stress [75,76]. Peroxiredoxins (PRDXs) are a group of ubiquitous enzymes (PRDX1-6) that have emerged as important and widespread peroxide and ONOO^−^ scavenging enzymes [77,78]. A growing body of evidence has begun to recognise the role of H_2_O_2_ in mediating redox signalling pathways during exercise, and thus it has been suggested that PRDX may be a salient regulator of exercise induced H_2_O_2_ levels (see review by Wadley et al. [79]).

## 4. Circadian Rhythms, Oxidative Stress, and Redox Homeostasis

**Figure 3:**
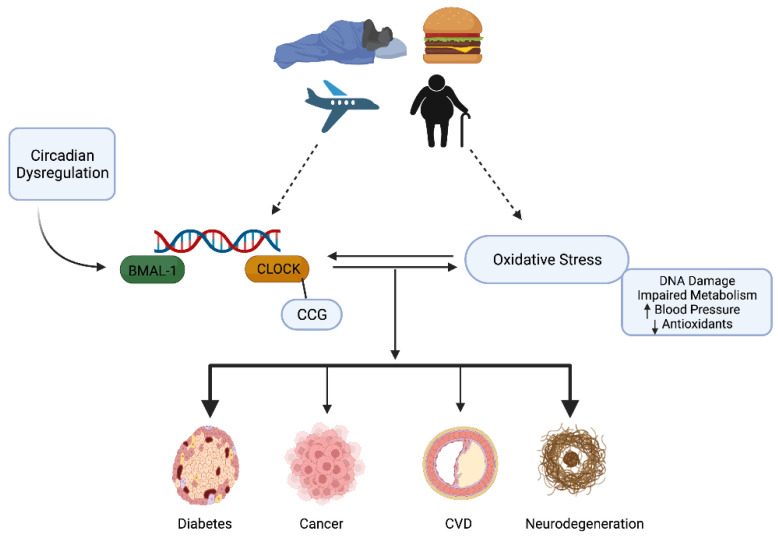
The interplay between Circadian and Redox Biology: Oxidative Stress and Disrupted Circadian Rhythms. A proposed bidirectional relationship exists between oxidative stress and circadian dysregulation whereby external factors and behaviours that disrupt circadian rhythms (jet lag, the ageing process, and regular consumption of certain foods) may also induce oxidative stress and, likely vice versa. The result is a deterioration of normal physiological functions (DNA damage, increased blood pressure, and insulin resistance) and control that increases the risk for chronic diseases.

Much is known about the cellular and molecular effects of RONS and circadian rhythms, respectively, but relatively less attention has been paid to the cross-talk and integration between the two and if/how these complex interactions affect physiological processes in health and disease. Current investigations have thus begun to focus on the molecular mechanisms linking RONS/oxidative stress and dysregulated circadian rhythms, sustaining the basis of a seemingly vital biological connection [80,81]—please see Figure 3. For instance, evidence now depicts a circadian influence on the immune system and this extends to the regulation of inflammatory and oxidative stress responses (especially in the ageing context—see review by [16]). It is now known that macrophages possess a molecular clock potentially able to impose temporal fluctuations in immune function [82]. RONS are integral to the proper functioning of the immune system and, interestingly, their production may be regulated by BMAL-1. In an elegant study by Early and colleagues [83] conducted on mice, ROS accumulation (detected by fluorescent probes) was increased in *BMAL-1^−/−^* macrophages, and related to decreased activity of Nrf2 in cells, resulting in a diminished antioxidant response (including a reduced synthesis of glutathione) and increased production of the proinflammatory cytokine, IL-1β. Conversely, the redox milieu may also modulate core clock regulation as the DNA binding activity of the CLOCK: BMAL1 heterodimer is dependent on the prevailing cellular redox status (as evidenced in the NADH/NAD^+^ and NADPH/NADP ratios), whereby binding is enhanced under reducing conditions [84]. Moreover, oxidative activation of Nrf2 via H_2_O_2_ has been shown to regulate core clock function by altering clock gene expression (*Per3*, *Nr1d1*, *Nr1d2*, *Dbp*, and *Tef*) and circadian function in mice in a comparable manner to overexpression or elimination of Nrf2 conditions [85].

### 4.1. Antioxidant Regulation and Control

Insight into the cross-talk between the circadian clock and oxidative stress has been gleaned from studies illustrating temporal expression patterns for antioxidant enzymes and compounds. For instance, O’Neill and Reddy [86] observed a circadian rhythm in mouse PRDXs that was altered in Cry1/2 double knockout cells, suggesting it may be regulated by the molecular clock. Using human RBCs, they also showed a robust ∼24-h redox cycle for PRDXs to directly show rhythms persist even without active transcription [86]. Furthermore, deletion of core clock components leads to increased thiol oxidation and protein carbonylation in flies and mice [87], and Per1-knockout flies exposed to oxidative stress display a shorter lifespan and increased oxidative damage compared to controls [88] which may, in part, be due to decreased activity of RONS scavenging enzymes. Given that RONS production can oscillate over the course of the day often due to normal, habitual behaviours, i.e., the by-products of metabolism, circadian control of antioxidants seems intuitive to maintain redox control [89]. Several studies have reported differences in DNA damage [90], lipid peroxidation [91], and protein oxidation [92] at different times of the day. These oscillations directly reflect the daily rhythm of antioxidant expression and protective enzyme activity levels. Those that peak in the morning includes: GPx; CAT; SOD; and PRDXs [6]. On the other hand, melatonin, plasma thiols, and ascorbic acid peak in the evening which also corresponds to the reported peaks for PERs1 and 2 and the CRYs (see [6]). Daily rhythmicity in SOD activity was first reported by Diaz-Munoz and colleagues in 1985. In contrast to humans, they found that in the rat cerebral cortex, SOD activity peaked in the dark phase, coinciding with the peak level of malondialdehyde (MDA), a marker of lipid peroxidation [93]. Thus, circadian oscillations in antioxidant expression seem to vary depending on the species investigated (i.e., nocturnal vs. diurnal), and this is most likely due to variations in sleep/wake cycles and the corresponding differences in their respective feeding patterns and light exposure [6].

The master regulator of antioxidant defence appears to be the transcription factor Nrf2, which translocates to the nucleus where it drives the expression of several antioxidant enzymes and is directly regulated by the expression of NRF through E box elements in the promoter [89]. As antioxidants are important for governing intracellular/local RONS levels, which, in turn, have been documented to impinge on the expression of clock genes, it appears that further appreciation and understanding of this cross-talk may yield purposeful applications for those interested in redox and chronobiology given how circadian rhythmicity and oxidative stress are central to many disease pathologies. For example, life-long administration of the antioxidant, N-acetyl-L-cysteine, has been shown to delay the onset of premature ageing induced by chronic oxidative stress in *BMAL-1^−/−^* mice [94].

### 4.2. Circadian and Oxidative Influence on Cardiovascular Physiology and Disease

Despite considerable progress in understanding, preventing, and treating cardiovascular diseases (CVD), ischaemic heart disease and strokes remain a major source of global morbidity and mortality [95]. Clock mechanisms are integral to normal cardiovascular function by coordinating rhythms in blood pressure, heart rate, and cardiac muscle contractility. However, circadian dysregulation increases cardiovascular risk with strong data suggesting adverse cardiovascular events such as sudden cardiac death [96] and myocardial infarction [97] are more likely to occur in the morning after awakening. Notably, a recent meta-analysis reported how the risk of acute myocardial infarction (AMI) increases after daylight saving transitions to emphasise the precarity of the circadian system [98]. The complete mechanism(s) surrounding such phenomena are unclear but may be linked to disruptions to redox control as RONS and free radical biology have been implicated in both the pathophysiology and treatment of heart disease [25]. For instance, we have reported increased peripheral arterial stiffness in healthy volunteers following the ingestion of a high-fat meal which was associated with augmented lipid hydroperoxides, while decreases in SOD were also observed [38]. We believe the high-fat meal increased O_2_^•−^ production that was subsequently able to react with endothelium-derived NO^•^ (via eNOS) to impair blood vessel function. The consequences of this (and similar reactions that elevate vascular RONS) may be detrimental to cardiovascular function by reducing NO^•^ bioactivity and increasing the formation of the ONOO^−^ [37], possibly leading to endothelial dysfunction and a pro-atherogenic environment [5]. ONOO^−^ interacts with lipids, DNA, and proteins via direct or indirect radical mechanisms, and its generation has been cited in the pathogenesis of stroke, myocardial infarction, atherosclerosis, circulatory shock, and chronic inflammatory diseases [99,100]. These RONS-instigated reactions trigger cellular responses ranging from subtle modulations of cell signalling to oxidative injury, committing cells to necrosis and apoptosis [100]. Of note, Man et al. [101] reported how the peripheral circadian clock can regulate eNOS and NO^•^ production (which is lower during the morning) and that lipid metabolism also displays circadian oscillations. Combined, the misalignment of the circadian clock with these parameters could lead to the development/progression of atherosclerosis, which may be heightened with frequent exposure to conditions that amplify RONS production such as high-fat meal ingestion, as discussed above. Support for a circadian-redox mechanism is further evident from a study in middle-aged adults where vascular endothelial function, as measured by flow-mediated dilation (FMD), was impaired across the night and into the morning period and was accompanied by a pronounced rise in plasma MDA and a concomitant augmentation in the vasoconstrictor, endothelin-1 (ET-1) [102]. Such interactions are clearly pertinent to those already predisposed to increased risk for cardiovascular events, and other diseases like cancer, such as shift workers.

### 4.3. Shift Work: DNA Damage and Repair

In a recent cross-sectional study, higher levels of H_2_O_2_ and lower SOD and catalase were observed in night workers when compared to day workers [103]. In fact, the circadian clock was first implicated as a factor in various diseases as epidemiological studies reported an increased incidence of cancers in long-term shift workers [19,104,105]. Those working night shifts also appear to have increased DNA damage that is linked to lower melatonin induced by night working [106]. In strict laboratory conditions, when compared to simulated day shifts for three consecutive days, simulated night shifts of the same duration also caused circadian dysregulation of genes involved in key DNA repair pathways. Moreover, the percentage of cells with BRCA1 and γH2AX foci (representing DNA damage biomarkers using immunofluorescent microscopy) was significantly higher in the night shift condition, whereas the effectiveness of the processes to repair leukocyte DNA damage from both endogenous and exogenous sources were compromised in samples from the night shift volunteers [107]. Given that it exerts antioxidant and DNA repair properties (via the nucleotide excision repair (*NER*) pathways), melatonin supplementation, acting as a so-called chronobiotic [32], has been suggested to reduce the potentially damaging effects of shift work [108]. The studies and data summarised in this section are undoubtedly useful for helping tie the molecular and biochemical connections between shift workers and elevated cancer risk, but much remains open for further discovery.

### 4.4. Diabetes, Obesity, and Metabolic Control

Normal CLOCK and BMAL-1 activity play a role in defending the body against metabolic disturbances, but CLOCK gene mutations are associated with hyperphagia, hyperlipidemia, hyperinsulinemia, hyperglycaemia, and sleep disorders—all of which are common in metabolic diseases such as diabetes and obesity [109]. Diabetes, especially Type 2 Diabetes (T2D), is connected to disruptions in normal circadian rhythms with shift-work, light pollution, jet lag, and increased screen time, all acting as potential contributory factors [33,110,111]. Emerging evidence has also identified melatonin, already known for its roles in circadian and redox physiology, respectively, as a potential mediator of glucose levels and insulin production [109] adding further credence to the existence of an important cross-talk between circadian rhythms and redox pathways in the regulation of metabolic control. Underpinning all forms of diabetes is either a decrease in β-cell mass or β-cell function [112,113,114]. Mechanistically, oxidative stress, potentially stemming from increased mitochondrial O_2_ production following excessive caloric intake and low activity levels, has also been implicated in diabetes as a key mediator of β-cell dysfunction and insulin resistance [5,115]. This appears to be secondary to circadian dysregulation as β-cells contain critical antioxidant genes that are targets not only of Nrf2, but also BMAL-1 which are vulnerable to circadian disruption in *BMAL-1^−/−^* mice [116]. Circadian disruption to these key antioxidants can thus lead to augmented β-cell mitochondrial RONS production in cells that already have relatively less antioxidant capacity, culminating in the impairment in β-cell function, insulin resistance, and diabetes [111,117].

Alterations to normal circadian control and oxidative stress are understood to be instrumental in the pathogenesis of other metabolic conditions associated with T2D, such as obesity [118]. Adipose tissue is a critical modulator of metabolic health, and oxidative stress can cause adipose tissue dysfunction by stimulating preadipocyte proliferation, adipogenesis, and chronic inflammation, which leads to obesity [119]. The circadian clock controls energy homeostasis by regulating circadian expression and/or activity of enzymes, hormones, and transport systems involved in metabolism with evidence showing that knockout and mutations in clock genes induce disruptions in adipose tissue function, differentiation, and metabolism (see review by Froy and Garaulet [120]). Disruptions to circadian control and oxidative stress may also affect other important metabolic organs. For instance, a relatively recent study found that night shift workers have a higher serum concentration of alanine aminotransferase (ALT) than daytime workers, indicating a potential association between circadian disruption and liver function [121] that could be important in the development of conditions such as non-alcoholic fatty liver disease (NAFLD). Moreover, such conditions proposed to be related to dietary factors (see review by Arrigo et al. [122]), are often accompanied by oxidative stress and seem to favourably respond to antioxidant and circadian therapy in the form of melatonin administration [123]. As alluded to, dietary factors and feeding patterns undoubtedly represent an important metabolic focus for their ability to modulate circadian function and redox control in health and disease, though further scrutiny is beyond the scope of this review.

Far from being fully determined, it does appear that a bidirectional relationship exists between RONS production and circadian rhythms: core clock function appears to be sensitive to changes in redox status and redox homeostasis may conversely be governed by clock machinery [124]. When out of sync, circadian dysregulations, oxidative stress, or both, can manifest. This is an intriguing frontier of research emerging, and the ability to harness these insights, combined with other important drivers of circadian and redox biology, will help to provide more focussed health and lifestyle prescriptions.

## 5. Exercise—Zeitgeber and Modulator of Redox Homeostasis

**Figure 4:**
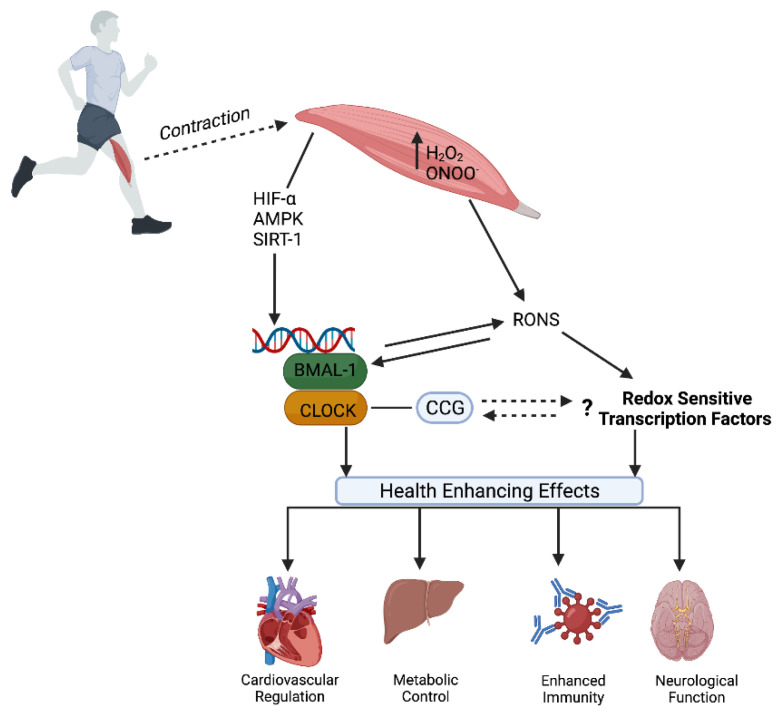
The interplay between Circadian and Redox Biology: Exercise as a zeitgeber and source of RONS. Exercise is a potent stimulus to entrain dysregulated circadian systems and positively affect the core molecular clock and subsequent expression of CCGs throughout the body. At the same time, exercise-induced RONS are integral to several recognised physiological responses and adaptations via the activation of redox-sensitive transcription factors (e.g., up-regulation of immunity, antioxidant enzymatic activity, etc.). It is possible that both circadian and redox signalling are inter-connected and operate synergistically to confer protective health effects following regular exercise.

Regular exercise is often regarded as one of the ‘best buys’ for public health given its multiple health-promoting benefits [125], with the Academy of Medical Royal Colleges [126] describing ‘the miracle cure’ of performing 30 min of moderate exercise, five times a week, as more powerful than many drugs administered for chronic disease prevention and management. Aerobic exercise training improves risk factors of metabolic syndrome such as glucose intolerance, hyperlipidemia, high blood pressure, low high-density lipoprotein content, and visceral obesity. Improved aerobic fitness increases neurogenesis, enhances memory, and may prevent brain atrophy [127]. Exercise is widely regarded for its substantial health benefits and there is now ample evidence from both observational studies and randomised trials to support that regular exercise is a contributing factor in the prevention of cardiovascular disease, cancer, diabetes, and other chronic conditions, as well as reducing the risk of all-cause mortality [128,129]. As more is understood regarding the molecular pathways and mechanisms through which the beneficial effects of exercise are transmitted, interest continues to grow in strategies to optimise the benefits of such effects and how these can be translated for optimal health and performance purposes (see Figure 4).

The circadian clock can be synchronised by photic and non-photic stimuli (temperature, physical activity, and food intake). Given that exercise represents a major challenge to whole-body homeostasis, provoking widespread perturbations in cells, tissues, and organs [130], modern theories have begun to probe the connections between exercise and circadian rhythms for both performance and health purposes. An extensive body of literature has established that exercise can influence the circadian system in rodents (see [1]) and emerging human evidence shows exercise can elicit phase-shifting effects which may be dependent on chronotype [34]. Exercise appears to be a potent entrainment factor for central as well as peripheral clocks, including those in muscle. For instance, the average core-clock gene expression (*BMAL1*, *ROR-α*, *CRY1*, *PER2*, *PER1*, and *NR1D1*) in male rugby players is significantly higher compared to sedentary males [131]. Therefore, the possibility of (a) exercise being able to attenuate the negative health effects of circadian misalignment and (b) the existence of an optimal time to exercise to maximise its therapeutic effects have become increasingly attractive to researchers and clinicians.

Of all the peripheral tissues, skeletal muscle represents a major downstream target for clock activity. Among other functions, elegant studies have shown how the molecular clock governs glucose metabolism in skeletal muscle. In one such study, *BMAL-1* deletion in mouse skeletal muscle manifested in impaired glucose uptake, reduced GLUT-4, and disrupted the activity of key glycolytic enzymes [132]. Thus, impairments in the muscle molecular clock appear to have important implications for the development of metabolic diseases such as T2D. As exercise is recommended for the prevention and treatment of T2D [9], it now appears that the beneficial metabolic effects exercise confers are, at least partly, achieved through actions on the muscle molecular clock to restore local circadian regulation [7]. For instance, skeletal muscle gene and protein expression of *BMAL-1* and *PER2* were increased in adults with obesity and pre-diabetes following 12 weeks of exercise training and this was accompanied by improvements in body composition, peripheral insulin sensitivity (glucose disposal rate), and maximal oxygen consumption. Specifically, *BMAL-1* gene expression correlated with glucose disposal rate [133].

While exercise undoubtedly elicits favourable, modulating effects in skeletal muscle metabolism, relatively little is known about the potency of these effects at different times of the day. This is important as the biological clock seems to drive patent rhythms in human skeletal muscle metabolism whereby mitochondrial oxidative capacity follows a day-night rhythm, peaking in the late evening and being lowest in the early afternoon [134]. As oxidative capacity is a vital determinant of exercise performance, it is unsurprising that studies report clear time of day effects (~10%) for exercise performance, capacity [8], and/or strength measures in human volunteers, which in one recent paper were correlated with *PER2* daily profiles [135]. The underlying mechanisms for such phenomena are complex but are thought to involve circadian fluctuations in core temperature, endocrine hormones, neuromuscular function, and metabolic flux [8,9]. One of the key contributing factors in these performance fluctuations is body temperature which can exert a myriad of regulatory effects on neuromuscular and metabolic activity and appears to peak in the late afternoon. As thermoregulation during exercise itself appears to follow a circadian rhythm, this may also explain the variability of fatigue onset when the same activity is performed at different times across the daily cycle, especially in longer, endurance-type exercise bouts [136]. Variations in human exercise efficiency (improved in Late vs. Early) have been ascribed to clock-driven fluctuations in metabolic control such as carbohydrate metabolism that requires lower oxygen consumption, a lower heart rate, and a lower rate of perceived exertion [8]. In competitive and elite sporting settings, this connection between exercise capacity and the molecular clock may be useful, to some extent, when planning to optimise training and competition schedules, but this is not an entirely new concept (see [137]).

Syncing exercise bouts, alongside other interventions not scrutinised in the current review (e.g., time-restricted eating), to align with circadian rhythms is an appealing paradigm to consider when trying to maximise the metabolic and health effects of exercise. In a crossover study involving 2 weeks of high-intensity interval training (HIIT), afternoon HIIT was more efficacious than morning HIIT at improving blood glucose in *n* = 11 men with T2D [138]. Such data indicate that the timing of exercise should be considered when prescribed for the management of T2D. However, in a larger study investigating the effects of exercise timing on glycaemic control in those with and without T2D, no distinct glycaemic benefits or alterations in circadian rhythm (as assessed by skin temperature) were detected between morning versus evening exercise [139].

Regular physical activity and exercise programmes have been shown to reduce the symptoms of patients with established CVD; additionally, prospective epidemiological studies of occupational and leisure-time physical activity have consistently documented a reduced incidence of CVD in the more physically active and fit individuals [140,141,142]. Consequently, exercise is regarded as an important intervention in tackling the burden imposed by CVD. Yet, the precise mechanisms by which exercise exhibits its ameliorating effects on the cardiovascular system still require elucidation. For instance, relatively little is known about the potential for exercise to entrain central and peripheral clocks and how this might influence cardiovascular health. It is conceivable that exercise may regulate circadian factors to partially influence cardiovascular health through the documented effects on skeletal muscle—given the connections between metabolic and cardiovascular health [5,7]. Studies have begun to explore this theme as well as the possible existence of diurnal effects of exercise on cardiovascular risk markers. Elevated arterial blood pressure, or hypertension, is a traditional cardiovascular risk factor that also forms one of the main constituents of metabolic syndrome [143,144]. Acute exercise can induce a transient reduction in blood pressure or post-exercise hypotension (PEH) [145]. This reduction is largely thought to be a result of increased blood flow to vascular beds and the subsequent decrease in the total peripheral resistance associated with exercise. The prolonged hypotensive effects of regular aerobic exercise may thus be due to repeated instances of PEH [146]. Despite this, BP reductions after aerobic training vary across studies and some factors, such as higher initial BP, moderate to high training intensities, and concomitant diet-induced weight loss, have been identified as promoters of a greater BP decrease [147]. Moreover, alterations to dietary composition (reduced fat and increased fibre) alongside exercise training have also been reported to decrease BP and oxidative stress markers in the absence of weight loss [148]. It is also possible that the time of day when aerobic training is performed may influence the extent of BP reductions after training. As most aspects of cardiovascular regulation demonstrate a circadian or diurnal pattern [149], it is plausible that the mechanisms driving sustained PEH are impacted by time of day. Some studies have tested this hypothesis with mixed results. In one study, the acute hypotensive effects following 30 min of steady state exercise were less marked in the morning versus the afternoon [150], but this may be masked due to the circadian effect on morning blood pressure [7]. While de Brito et al. [151] demonstrated that aerobic training performed in the evening decreased clinic and ambulatory BP when compared to morning training in hypertensive men, this finding may be explained by the use of antihypertensive medications in the morning group. In contrast, while aerobic exercise performed both in the morning and the afternoon/evening contributed to PEH when circadian influences of morning blood pressure were considered, PEH was greater following morning exercise rather than evening exercise in a separate study [152]. The influence of exercise intensity and other zeitgebers like food ingestion, light exposure, and sleeping patterns are complex confounders in such studies, but exercise may induce re-alignment of the circadian clock and better cardiovascular outcomes by modulating hormonal responses and heart rate (see review by [7]).

It is well established that aerobic exercise can improve endothelial function and flow-mediated dilation (FMD) responses [153], particularly in those with cardiovascular disease and related risk factors [154]. Despite this link, the diurnal effects of exercise on FMD have received relatively little attention, even though FMD has been shown previously to vary with time of day [155]. A handful of studies have nevertheless provided useful insights to illustrate the apparent diurnal effects of exercise on FMD in some [156,157] but not all instances [152]. Although beyond the scope of this paper, the equivocal findings in such studies may be partially explained by variations in the exercise stimuli used e.g., whole body versus isolated limbs.

Endurance exercise training elicits a number of benefits such as increased skeletal muscle mitochondrial number and volume density leading to improved oxidative capacity [158]. An important development in unraveling the cellular events that promote mitochondrial biogenesis and other beneficial effects such as angiogenesis, improved antioxidant defences, and enhanced fat metabolism [11] was the discovery of the transcriptional coactivator PGC-1α, thought to be the master regulator of the process. Recently, this exercise-stimulated pathway has been identified as being downstream of the molecular clock, providing a molecular mechanism through which circadian timing can influence exercise responses [12,159]. In recent years, the understanding of this mechanism has grown to the extent that the pathway relies on redox signals, whereby exercise-induced RONS appear to potentiate PGC-1α and NF-kB [48]. Evidence to support this supposition comes from studies such as that from Ristow and Colleagues [53] who showed the use of a vitamin C and vitamin E antioxidant regime blunted mRNA responses in several markers of mitochondrial biogenesis, including PGC-1α, in healthy young men completing a training intervention. Mechanistically, PGC-1α regulation at rest and during exercise depends on several factors with evidence suggesting that AMPK, Nrf2, and p38 serve as the principal intermediate molecules connecting RONS with PGC-1α activation and/or mitochondrial biogenesis markers [57]. Redox activation of Nrf2 has already been identified in the priming of the antioxidant network [83] and in the regulation of core clock function through its activation of clock machinery [85]. Moreover, exercise-induced RONS and redox-sensitive genes and transcription factors, including AMPK, HIF-1α, and PGC-1α, may influence the expression of core molecular clock genes [11,12]. Given the cross-talk between HIF pathways and circadian clock control [27], and that RONS are thought to be instrumental in the exercise-induced increases in HIF-1α [57], HIF-1α may be a crucial mediator connecting circadian control, redox homeostasis, and exercise.

While the majority of redox-focussed exercise studies have examined aerobic/endurance training, a growing appreciation for the role of RONS signalling in resistance training-mediated adaptations have been outlined. In particular, the mechanistic target of rapamycin complex 1 (mTORC1) activation, which stimulates protein synthesis via increased translation of contractile protein mRNA central to muscular hypertrophy, has shown to be redox-sensitive. For example, and as previously highlighted, ONOO^−^ has been implicated in the signal transduction pathways for mTORC1 that lead to hypertrophy [67]. As mTORC1 appears to exhibit diurnal oscillations—current evidence has highlighted the suppressive effect of PER2 on mTORC1 [160], the extent to which these are influenced by RONS signalling and if/how this might be integral to diurnal exercise responses remains uncovered and ripe for investigation.

## 6. Future Directions

Contemporary research has identified circadian disruptions to augment oxidative stress and subsequently aberrate adipose tissue function and metabolism. Therefore, circadian machinery in the adipose tissue may be a novel therapeutic target for the prevention and treatment of metabolic and cardiovascular diseases [119]. We believe that exploratory exercise studies connecting circadian principles to adipose tissue and redox metabolism may represent one such approach. Based on the existing literature, there appears to be scope for mechanistic and maybe even clinical progress, but it is imperative that future investigations are carefully designed to control for confounding factors including participant chronotype and the influence of other zeitgebers [34] and testing of insufficient numbers of volunteers in a narrow range of times across the 24 h day [1]. Importantly, new studies could also benefit from the careful selection of appropriate methods to measure and assess the impact of potential interventions on circadian rhythms [161]. Exercise stimuli and protocols such as intensity, mode, etc. should also be carefully chosen when considering the potential implications for biological rhythms and RONS. Some of the equivocal findings on the health/physiological effects of exercise timing described in the review may be due to the heterogeneity of the volunteers. More parallel investigations are warranted for this potentially promising realm of exercise prescription research, such as those that incorporate larger sample sizes, crossover study designs, and, crucially, chronic exercise interventions that assay a range of clinical and mechanistic redox markers. Of course, as alterations in feeding patterns and diet can affect circadian rhythms and redox markers [119,162], further mechanistic investigations are also welcome that seek to simultaneously explore dietary manipulations with exercise to aid in the development of targeted treatment strategies to improve health in those with/or at risk from chronic disease.

## 7. Conclusions

Roenneberg and Merrow [19] offer a plan on how to implement chronobiological principles into medicine through the identification of a mechanism that determines the circadian clocks of an individual and their ability to be synchronised. This knowledge could inform the prescription of specific exposures of zeitgebers to target the circadian system and personalise the therapeutic schedules for any individual [109]. Targeting circadian health would be especially pertinent to those at risk from circadian misalignment from shift work, frequent bright light exposure, sleeping disorders, and frequent travel across multiple time zones. We believe that exercise might represent one such zeitgeber given its ability to potentially entrain the human circadian system; additionally, careful interrogation of how it may modulate the cross-talk between the molecular clock and redox biology could yield further insights into the salient mechanistic pathways such as those that control antioxidant defences and DNA repair. At present, and despite the promise of studies showing diurnal variations in exercise responses/adaptations, much more work is needed to comprehensively identify whether an optimal exercise time exists. As it stands, exercise prescription should be personalised based on several variables such as the individual’s health and fitness status (i.e., the absence or presence of CVD risk factors), chronotype, work and domestic patterns (including mealtimes and work schedules), and preferences. Given the recent advances in redox and molecular biology, informed by advancements in analytical techniques, exercise studies positioned at the emerging nexus between circadian rhythms and the redox milieu represent a fascinating area for exploration, especially for those that advocate, or wish to scrutinise, exercise prescription for health.
